# Spinal Cord Changes After Laminoplasty in Cervical Compressive Myelopathy: A Diffusion Tensor Imaging Study

**DOI:** 10.3389/fneur.2018.00696

**Published:** 2018-08-29

**Authors:** Young-Mi Yang, Woo-Kyoung Yoo, Shahid Bashir, Jae-Keun Oh, Yoon-Hae Kwak, Seok Woo Kim

**Affiliations:** ^1^Spine Center, Hallym University Sacred Heart Hospital, Anyang, South Korea; ^2^Department of Physical Medicine and Rehabilitation, Hallym University Sacred Heart Hospital, Anyang, South Korea; ^3^Department of Neurophysiology, Neuroscience Center, King Fahad Specialist Hospital Dammam, Dammam, Saudi Arabia; ^4^Department of Neurology, Berenson-Allen Center for Noninvasive Brain Stimulation, Beth Israel Deaconess Medical Center, Harvard Medical School, Boston, MA, United States; ^5^Department of Neurosurgery, Spine Center, Hallym University Sacred Heart Hospital, College of Medicine, Hallym University, Anyang, South Korea; ^6^Department of Orthopaedic Surgery, Hallym University Sacred Heart Hospital, Anyang, South Korea

**Keywords:** spinal plasticity, neuroimaging, spinal cord injury, spine surgery, functional recovery

## Abstract

**Purpose:** Validation of the efficacy of decompression surgery in patients with cervical myelopathy (CM) is important in terms of the recovery of the integrity of the spinal cord. However, to date, no longitudinal study has addressed the underlying pathological changes using diffusion tensor imaging (DTI) in CM patients. This study aimed to determine the diffusion metrics at the lesion as well as below the lesion level longitudinally in CM patients following laminoplasty using DTI.

**Methods:** Twenty CM patients were analyzed and compared with 20 age-matched healthy controls. The primary outcome measure was the changes in the diffusion metrics [fractional isotropy (FA), mean diffusivity (MD), axial diffusivity (AD), and radial diffusivity (RD)]. The secondary outcome measure was the changes in the modified Japanese Orthopedic Association (mJOA) score. Diffusion metrics obtained from six region-of-interests (ROIs; 2 anterior, 2 posterior, 2 lateral) at the lesion and below the lesion level (C7/T1) in preoperative and 6 months postoperative conditions were compared longitudinally.

**Results:** The CM patients showed significant changes in their postoperative diffusion metrics for the anterior ROIs compared with the preoperative measures both at and below the lesion level. In the lateral and posterior cord, the preoperative AD value decreased after laminoplasty to the control at the lesion level. In contrast, MD and RD values at the lesion level and FA value at below the lesion level remained unchanged postoperatively. In addition, the postoperative anterior FA value was positively correlated with the postoperative mJOA score below the lesion level.

**Conclusion:** This would be the first study showing changes in the spinal cord at the lesion as well as below the lesion level after laminoplasty in CM patients, which may be associated with functional recovery.

## Introduction

Cervical myelopathy (CM) is a common age-related disorder that presents symptoms of unsteady gait, worsening balance, clumsy hands, spasticity, and voiding difficulty, which often progressively leads to motor paralysis and sensory disturbances (1).

Currently, magnetic resonance imaging (MRI) plays an essential role in the diagnosis and follow-up of spinal cord lesions. However, unfortunately, there is a substantial disagreement about the discrepancy between MRI and clinical findings (2, 3, 4, 5). One of the reasons about the discrepancy between MRI and clinical findings is suggested by an asymptomatic period that could last for many years, which means that potential changes have taken place in the spinal cord as a result of adjusting to pathological changes during that period (6, 7). Although validation of the efficacy of decompression surgery in CM patients has always been a matter of great interest regarding the recovery of the spinal cord integrity; it has not been tackled sufficiently due to the technical difficulties in assessments of those pathological changes.

Recent advances in the diffusion tensor imaging (DTI) technology have enabled the measurement of white matter fiber integrity both quantitatively and qualitatively. Several studies have previously reported on the diagnostic and predictive value of spinal cord DTI in CM (3, 4, 8–10). DTI analysis is based on the diffusion properties of the water molecules, which can be presented in a diffusion metrics that shows the directionality in a relative value between 0 and 1 (FA) (11), and the total amount of diffusivity (MD), which is the sum of the axial (AD), and horizontal diffusivity (RD). The FA value is generally considered closely related to the function (12) and decreased FA could implicate a reduced number of fibers or a reduced density results in increased extracellular space (13).

The MD value is thought to be useful for monitoring the pathological condition, such as the progression of recovery from spinal cord injury (14, 15).

Most of those studies have consistently shown that the DTI metrics sensitively reflected the pathological status and were correlated with the behavioral outcome (2–5). However, as myelopathy symptoms are closely associated with severity of the damage to the axonal integrity of the descending tract below the lesion, it is essential to measure the damage in the distal portion below the lesion for an evaluation of the prognosis and outcome. Nevertheless, most of them are focused on the lesion site; only one longitudinal study (16) has addressed the pathological changes without detailed information of the diffusion metrics.

To the best of our knowledge, this study would be the first study to compare changes longitudinally between various preoperative and postoperative diffusion metrics, especially in the distal part of the lesion as well as the lesion site in CM patients. Therefore, we aimed to determine whether there are changes in the spinal cord of CM patients both within the lesion and intact below the lesion following laminoplasty, to elucidate the recovery mechanism in CM. We hypothesized that laminoplasty would promote the recovery from the myelopathic condition, and that this will be confirmed by changes in diffusion metrics and functional recovery accordingly.

## Materials and methods

### Participants

Thirty patients, who were diagnosed with CM (mean age: 52.8 ± 8.0 years, Male:Female = 17:3, Table 1), were initially recruited between September 2010 and August 2012. Patients who met the following eligibility criteria were included in the study: (1) patients aged 18 years or older and above (due to full mature ossification); (2) patients with history of clinical symptoms with compressive lesions (cervical spondylotic myelopathy, ossification of the posterior longitudinal ligament and the ligament flavum, cervical disc herniation, and cervical spinal stenosis) as visualized on T2-weighted MRI; and (3) patients who underwent laminoplasty. The patients' exclusion criteria were as follows: (1) previous spine surgery, (2) rheumatoid arthritis, (3) spine diseases, including tumors, spinal infection, trauma, instability, and neurological disorders other than spinal origin, and (4) congenital anomaly of the spine and CM patients who had severe kyphotic curvature of the cervical spine of >15°. Among these patients, six patients who had surgery with hardware and four patients who had previous lumbar surgeries were excluded. Finally 20 patients of CM who met the eligible criteria were included in the study.

**Table 1:** Baseline characteristics of the CM patients.

**Age range (years)**	**M/F**	**Post-op. f/u time (day)**	**Lesion level**	**Stenosis**	**Signal change**	**Compression ratio (%)**	**mJOA score**	
							**Pre-op**	**Post-op**
35–40	1/0	190.0	C4-5 (1)	100%	100%	37.4	12.0	13.0
41–45	1/1	201.5	C4-5 (1) C5-6 (1)	100%	100%	22.2	15.5	16.0
46–50	3/0	185.0	C3-4 (2) C5-6 (1)	100%	66.7%	32.2	16.3	16.7
51–55	2/2	201.8	C3-4 (2) C5-6 (2)	100%	100%	28.0	12.8	14.0
56–60	5/0	180.0	C3-4 (2) C5-6 (2) C6-7 (1)	100%	100%	28.5	14.2	14.8
61–65	–	–	–	–	–	–	–	–
66–70	5/0	192.4	C3-4 (2) C4-5 (2) C5-6 (1)	100%	80%	51.8	14.4	15.4
52.8 ± 8.0	17/3	190.9 ± 17.2		100%	90%	34.6 ± 15.6	14.5 ± 1.7	15.3 ± 2.0

*M, male; F, female; f/u, follow up; mJOA, Modified Japanese Orthopedic Association*.

Twenty age-matched healthy controls (mean age: 54.7 ± 9.1 years, Male:Female = 15:5) with normal conventional spine MRI were recruited. None of these controls had a history of psychiatric or neurological conditions, and all had normal medical and neurological results.

The study protocol was approved by the local Institutional Review Board (IRB, 2010-I057), which is registered in the Clinical Research Information Service (CRIS, KCT0001331). All participants provided their written informed consent to participate before enrolling in the study, which had been approved by the local Institutional Review Board and was conducted in compliance with the Declaration of Helsinki.

### Patient operations

As laminoplasty is known as one of the most common surgical treatments of CM and it involves enlarging the spinal canals with or without the use of any metallic device, In every single case involved in our study, spinal canal enlargement was achieved by opening the split laminae bilaterally with a spreader and placing allo-bone graft (Laminar Spacer-K; CG Bio, Seoul, Korea) to prevent possible interference of the metal instrument on the MRI after bilateral gutters for the hinges were carefully created with a high-speed burr at the transitional area between the facet joint and the laminae. All of the laminoplasty operation was performed by one operator.

### Cervical myelopathy severity measure

All the CM cases were carefully evaluated, and their neurological status, including motor and sensory changes, was recorded. Symptoms were also evaluated according to their severity, as measured by the modified Japanese Orthopedic Association (mJOA) score, at two-time point, preoperative and 6 months postoperative. The mJOA score is the most commonly used clinical measure of the severity of spondylotic myelopathy, which is composed of four groups of functions: arms, legs, micturition, and sensory capacity of the hands (17, 18). Mid myelopathy can be defined as mJOA score from 15 to 17, moderate as mJOA from 12 to 14 and severe as mJOA from 0 to 11.

### DTI acquisition and analysis

T2-weighted images were obtained using a 3-T Achieva MRI scanner (Achieva, Philips, Best, The Netherlands), with the following parameters: the same orientation plane as for diffusion-weighted data (axial), a slice thickness of 3 mm, no gap, time repetition/echo time 3,600/120 ms, and field of view of 250 mm. DTI was obtained using single-shot echo-planar imaging with the following parameters: b value of 500 s/mm^2^, number of diffusion gradient directions 15, slice thickness 3 mm, no gap, time repetition/echo time 6,300/63 ms, and acquisition matrix 128 × 128. T2-weighted MRI and DTI were obtained at the two time points, at preoperative and at a 6 months follow-up after laminoplasty.

One investigator who was blinded to the clinical conditions conducted the DTI analysis. Briefly, the raw image files were preprocessed using FSL software (University of Oxford, Oxford, UK) and then co-registered into T2-weighted MRI using the SPM8 software (University College London, London, UK). DTIFIT implemented in FSL software were used to obtain the DTI parameters, including the fractional anisotropy (FA), mean diffusivity (MD), axial diffusivity (AD), and radial diffusivity (RD) values.

For the precise comparison of the preoperative and postoperative DTI values, it was ensured that the head and neck of each participant were checked at the same position by the same radiologist during both MRI acquisitions. At least two investigators in our spine center confirmed each region-of-interest (ROI) using both T2 images and color-coded FA maps. The ROIs of each half of the spinal cord in the anterior, lateral, and posterior white matter regions on the “H-shaped” gray matter were marked at the lesion level and the C7/T1 intact level in the cervical vertebrae. Thus, six ROIs were defined at each of these two vertebral levels, for a total of 12 (4 anterior, 4 lateral, and 4 posterior) ROIs in each participant according to the method described by Yoo et al. and Muller-mang et al. (4, 19). The values of FA, MD, AD, or RD for a given ROI were extracted and averaged in the groups for comparison. The mean postoperative follow-up time was 190.9 ± 17.2 days (range of 175–249 days).

### Statistical analysis

The paired-sample *t*-test of the SPSS 20.0 statistical package (SPSS, Chicago, IL, USA) was used for preoperative and postoperative condition comparisons in CM patients. The independent-samples *t*-test was used for a comparison of the DTI metrics between the CM patients and controls. After Bonferroni correction for multiple comparisons (0.05/48 ≈ 0.00104), *p* < 0.001 was regarded as a significant difference in the comparison between controls and CM and pre and postoperative condition in each DTI indices. The Pearson bivariate correlation was determined between all of the DTI metrics and the mJOA scores at both the preoperative and postoperative conditions (*p* < 0.05).

## Results

### Baseline characteristics of the CM patients

The characteristics of 20 CM patients and the compression sites, including the myelopathy evaluations, are shown in Table 1. The CM patients had a mean compression ratio of 34.6 ± 15.6% as measured according to the method described by Fujiwara et al. (20). All of the CM patients showed signal changes in the MRI at the lesion level except for two cases. The mJOA score showed moderate in 11 and mild impairment in 9 patients (Table 1) (21).

### Primary outcome: changes in the diffusion metrics before and after surgery

#### Comparison of the diffusion metrics at the lesion level

**Figure 1:**
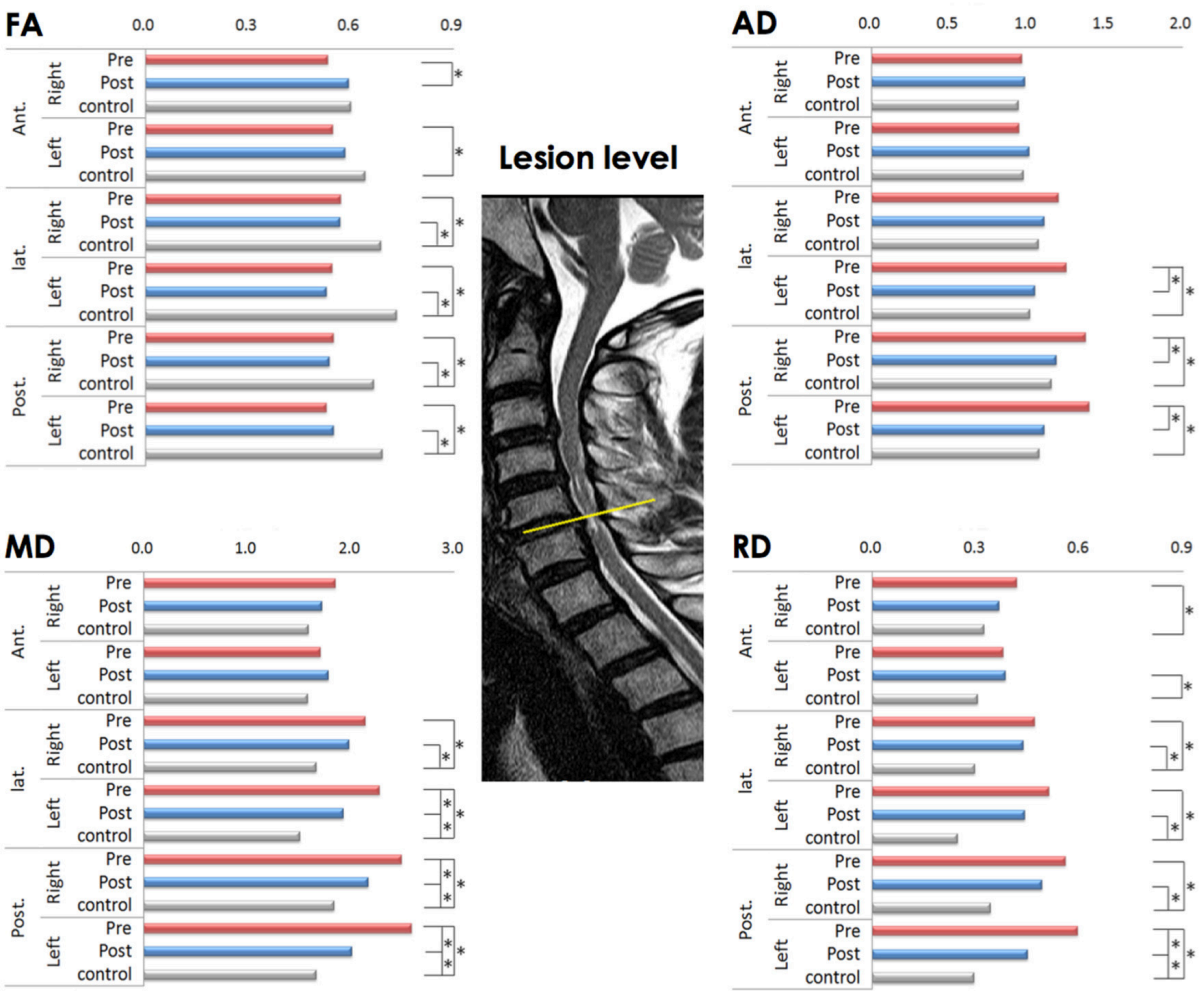
Comparison of diffusion metrics at six regions of interest (ROIs) at the lesion level before and after laminoplasty (matched with healthy controls). FA (fractional anisotropy), MD (mean diffusivity), AD (axial diffusivity), and RD (radial diffusivity) values; * *p* < 0.001 is considered to be statistically significant; the yellow line indicates the lesion level.

On the preoperative condition, the FA of the left anterior, bilateral lateral and bilateral posterior ROIs decreased compared to controls. This decrease in FA remained significant in the bilateral lateral and posterior ROIs. The significant increase of FA on the postoperative compared to preoperative condition noted in the right anterior ROI (Figure 1).

The significant increase of MD observed in the left lateral and left posterior ROIs in comparison to controls on the preoperative condition, which remained significant only in the left lateral ROI on the postoperative condition. There was no significant difference between preoperative and postoperative conditions in MD (Figure 1).

The AD significantly increased in the left lateral and left posterior ROIs on the preoperative condition compared to controls, which was reduced to not significant on the postoperative condition (Figure 1).

The RD significantly increased in the right anterior, left lateral, and both posterior ROIs on the preoperative condition compared to controls. This increase of RD left to be significant in the left lateral and left posterior ROIs on the postoperative condition (Figure 1).

#### Comparison of diffusion metrics below the lesion (C7/T1) level

**Figure 2:**
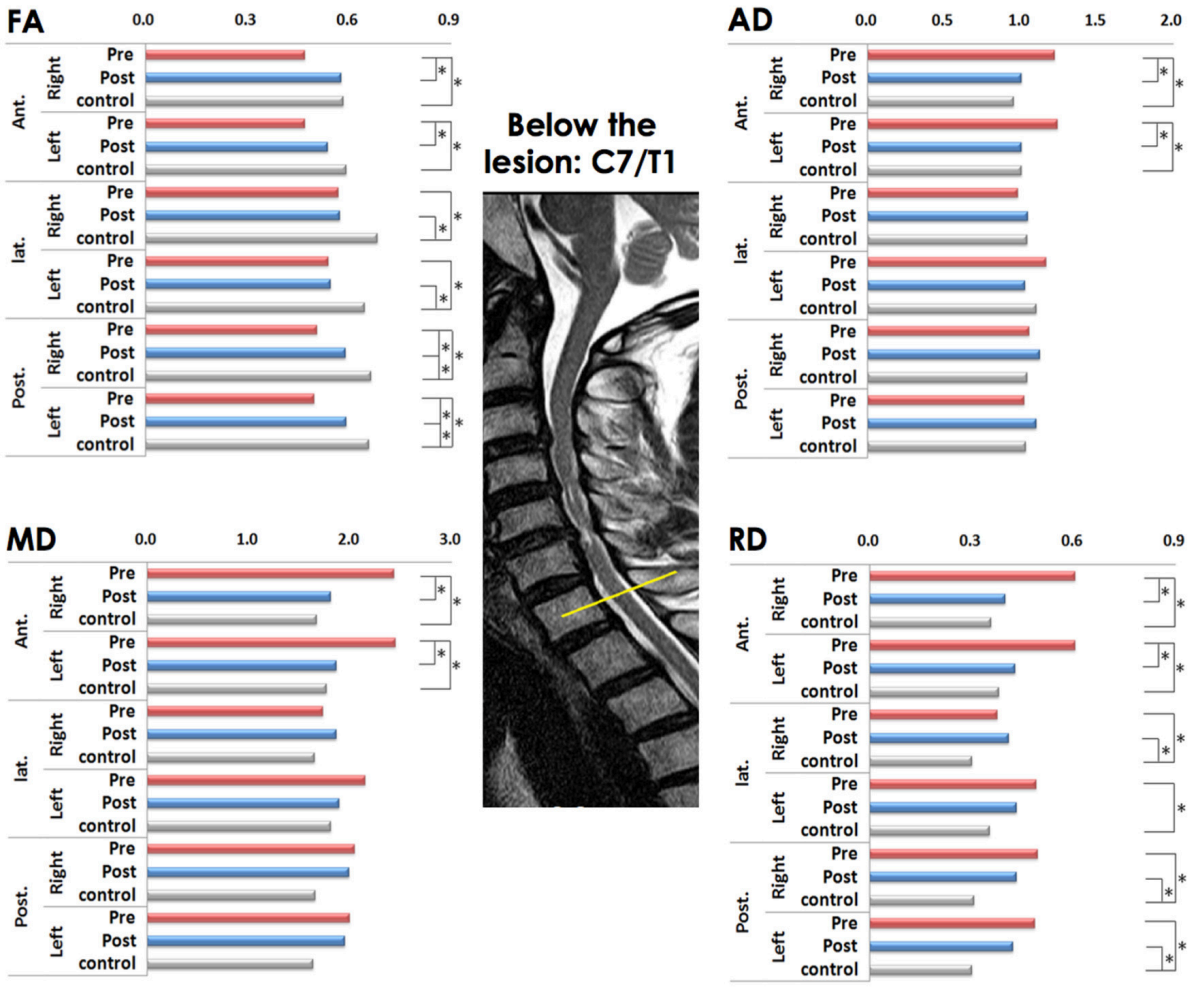
Comparison of diffusion metrics at six regions of interest (ROIs) below the lesion level before and after laminoplasty (matched with healthy controls). FA (fractional anisotropy), MD (mean diffusivity), AD (axial diffusivity), and RD (radial diffusivity) values; * *p* < 0.001 is considered to be statistically significant; the yellow line indicates below the lesion level.

On the preoperative condition, the FA of the all ROIs decreased compared to controls. After laminoplasty, the FA in the bilateral anterior ROIs showed no statistical difference compared to controls with increased postoperative FA value. In comparison of preoperative and postoperative conditions, there was significant difference in FA of right anterior ROI. However, in the right lateral and bilateral posterior ROIs remained to be decreased significant postoperatively (Figure 2).

The MD increased only in the right anterior ROIs compared to controls in preoperative condition (Figure 2).

There was no significant difference compared between preoperative and postoperative conditions in MD and AD (Figure 2).

The RD also was increased in all ROIs compared to controls in preoperative condition, which left significant in the left posterior ROI on the postoperative condition. Among them, only right anterior ROI was significantly decreased postoperatively (Figure 2).

### Secondary outcome

#### Comparison of the mJOA scores before and after surgery

The mean mJOA score significantly improved in the postoperative (15.3 ± 2.0) compared with the preoperative condition (14.5 ± 1.7; *p* = 0.002). The level of severity in mJOA score decreased in eight patients among whom four improved from moderate to mild severity, while the other four improved from mild to normal. Five patients showed an improved mJOA scores without changes in the severity category, while one patient presented with a decreased mJOA score in the follow-up evaluation (Table 1).

#### Correlation analysis

**Figure 3:**
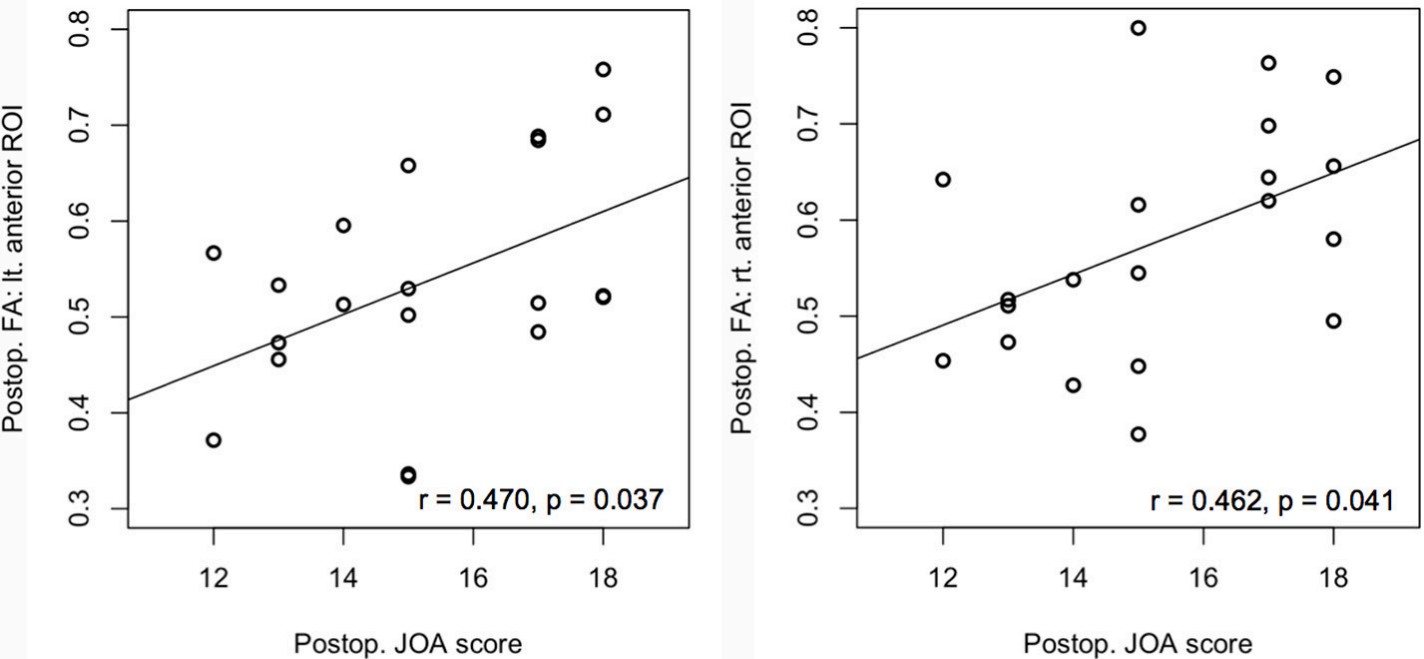
Correlation of the preoperative and postoperative FA values with the preoperative and postoperative mJOA scores at the anterior regions of interest (ROIs) below the lesion level (C7/T1). Significant correlations were observed only between postoperative anterior ROIs with the postoperative mJOA scores. FA, Fractional anisotropy; mJOA, Modified Japanese Orthopedic Association, *p* < 0.05.

There was no significant correlation between the diffusion metrics and mJOA at the lesion level. In contrast, the postoperative FA of both anterior ROIs at below the lesion level correlated with the postoperative mJOA score (left side: *r* = 0.470, *p* = 0.037; right side: *r* = 0.462, *p* = 0.041) (Figure 3).

## Discussion

This study would be the first study to show a decompression effect of laminoplasty in CM patients by comparing preoperative diffusion metrics with the postoperative ones at the lesion and below the lesion level, which showed significant changes in the right anterior cord that were reversed by laminoplasty. In addition, both postoperative anterior FAs were positively correlated with the postoperative mJOA score below the lesion.

Recent advances in DTI facilitated the investigation of white matter fiber integrity of the spinal cord *in vivo*. Typically, CM is caused by chronic events, but acute events combined with chronic segmental compression of the spinal cord with degenerative changes may also cause CM. Wallerian degeneration usually begins to develop toward the distal part of an injured neuron after axonal injury, and can be detected even at distant sites from the lesion in animal models (22). Because myelopathy symptoms are closely associated with severity of the damage to the axonal integrity of the descending tract below the lesion, it is essential to measure the damage in the distal portion below the lesion for an evaluation of the prognosis and outcome; however, most of the previous cross-sectional studies have only examined only the lesion level or the C2-3 level for the control (2, 10, 23, 24). In this study, we obtained diffusion metrics at the lesion level as well as below the lesion at the C7/T1 level.

The findings obtained at the lesion level showed significant changes in FA and RD in most studied ROIs both in preoperative and postoperative conditions compared with controls. Thus, at the lesion level, the integrity of the spinal cord improved only in comparison with controls after laminoplasty, as indicated by increased FA and decreased MD, AD, and RD values. These results are consistent with previous reports describing changes in the diffusion metrics in CM patients compared with controls (10). When we analyzed the diffusion metrics by dividing each region at the lesion level, we observed a decrease in the AD similar to controls, while RD value decreased but left significant in the left lateral and left posterior ROIs. This suggests possible changes in axonal integrity in the acute phase has been regained its integrity due to reduced extra-axonal diffusion restrictions along the longitudinal axis caused by an increase in the partial volume of the cell bodies within the tract (25) with remained decreased radial diffusivity, which might considered to have myelinopathy as the reduction in the radial diffusion might be caused by the breakdown of the myelin sheath. In contrast, in the anterior cord, decreased FA and no changes in AD along with increased RD was observed, which implicating a myelinopathy condition in the preoperative condition that was partially reversed following laminoplasty. At the lesion level, the injury was more severe in the posterior spinal cord compared with the anterior spinal cord, while it improved partially in both anterior and posterior portions postoperatively.

In contrast, because there are no significant structural changes below the lesion level, measuring changes in this level would have important clinical significance for the estimation of recovery. In this study, all of the diffusion metrics in the anterior portion of the spinal cord showed significant changes below the lesion level postoperatively. Notably, FA was increased, and MD was decreased, which was mostly influenced by RD following the decompression surgery. Thus, these findings could be interpreted as a reversal of a myelinopathy condition following laminoplasty. In the lateral and posterior cord, the preoperative AD value decreased significantly after laminoplasty to the control at the lesion level. In contrast, MD and RD values at the lesion level and FA value at below the lesion level remained unchanged postoperatively. These findings might implicate that there would be a myelinopathy present even after the decompression surgery.

There have been few studies reporting on longitudinal changes following decompression surgery in CM patients (16, 26). None of these studies have presented measurements of the diffusion metrics below the lesion level. One study measured the FA values at the C6-7 level in the preoperative condition, and the results were well correlated with the functional scores (20). The value of our findings lies in particular in the fact that our measurements were obtained longitudinally from the same individuals following laminoplasty. FA decreases below the lesion level have been previously reported in patients with chronic spinal cord injuries, where the decreases were greater in cases of complete injury compared with an incomplete injury (14). Most of the patients with incomplete spinal cord injury in that study showed more motor dysfunctions than sensory impairment. Considering that disruption and Wallerian degeneration would be greater in the motor tracts, such as the corticospinal tract, compared with sensory tracts and that abnormal FA values would be present in a descending pattern rather than in an ascending pattern (27), our results that showed changes in the diffusion metrics and a correlation with motor scores only in the anterior ROIs clearly support those findings.

There are several limitations to this study that are worth noting. First, there might be technical problems arising from severe compression. This might have made it difficult to distinguish the ROIs at the lesion level of the spinal cord, despite measured the ROIs in detail and voxel by voxel (23). In addition, although we strictly controlled the neck position of each participant in the scanners between MRI acquisitions, it might not be easy to get a perfectly matching image in the process of taking MRI and analysis of DTI with same patients during the follow-up. A second possible limitation is the surgical factor, which would have increased the local extracellular edema in specific areas. Although those changes are part of the pathological findings that we measured, the findings at the level of the lesion, where there are certain amounts of structural derangement due to compression, should be cautiously interpreted. Third, the significant diffusion metrics changes we observed in the lateral and posterior ROIs at and below the lesion level could not be correlated with sensory symptoms since the mJOA score does not represents the specific sensory function. Lastly, the point that there was no other comparable neurophysiological measure shown in this study for the validation of the integrity of the spinal cord would be a limitation, which warrant further study in the future.

Nonetheless, based on our observations obtained longitudinally from the same individuals following laminoplasty, this study has shown that the DTI metrics sensitively reflected the pathological changes, which were correlated with the clinical findings in the spinal cord of CM patients both within the lesion and below the lesion after surgery.

## Conclusions

This study is the first to show changes in the spinal cord both at and below lesion level after laminoplasty in CM patients, which may be associated with functional recovery.

## Author contributions

SK: conception and design, acquisition of data, analysis and interpretation of data, drafting of the manuscript, critical revision of the manuscript for important intellectual content, statistical analysis, obtaining funds, supervision, administrative, technical or material support. Y-MY: acquisition of data, analysis and interpretation of data, statistical analysis, administrative, technical or material support, obtaining funds. W-KY: acquisition of data, analysis and interpretation of data, statistical analysis, critical revision of the manuscript for important intellectual content, statistical analysis, obtaining funds. SB: analysis and interpretation of data, critical revision of the manuscript for important intellectual content, statistical analysis, obtaining funds, administrative, technical support. J-KO and Y-HK: analysis and interpretation of data, statistical analysis, critical revision of the manuscript, administrative support.

### Conflict of interest statement

The authors declare that the research was conducted in the absence of any commercial or financial relationships that could be construed as a potential conflict of interest.
